# Supplementary material for: Reverse genetics in humanized mice reveals CARD8-mediated pyroptosis causing pancytopenia in human DPP9 deficiency
Source: bioRxiv. 2026 Jun 30:2026.06.27.735024. Preprint. [Version 1] doi: 10.64898/2026.06.27.735024 (PMC13345154; doi:10.64898/2026.06.27.735024)

bioRxiv preprint doi: https://doi.org/10.64898/2026.06.27.735024; this version posted June 30, 2026. The copyright holder for this preprint (which was not certified by peer review) is the author/funder, who has granted bioRxiv a license to display the preprint in perpetuity. It is made available under a CC-BY 4.0 International license.

Supp Fig. 1. (**A**) Markers of iPSCs following hematopoietic stem cell differentiation following manufacturer's protocol (Stemcell technologies). (**B**) Neonatal engraftment of iPSC-derived hematopoietic stem cells in MISTRG6 mice. $hCD45^{+}$, human $CD45^{+}$. (**C**) primary CD34+ human HSPCs are electroporated with varying quantity of Cas9 and sgRNA against the TRAC locus. PCR amplicon around the edited region. L: Ladder. (**D**) Inferred Editing Efficacy (Synthego) based on Sanger traces from samples in panel C). (**E**) Sanger traces around the *TRAC* locus around sgRNA 1 cut site (g1), sgRNA 2 cut site (g2), sgRNA 3 cut site (g3). (**F**) $CD34^{+}$ human HSPCs are cultured overnight in X-VIVO 15 or SFEM media and the number of cells recovered the next day is quantified. (**G**)

bioRxiv preprint doi: https://doi.org/10.64898/2026.06.27.735024; this version posted June 30, 2026. The copyright holder for this preprint (which was not certified by peer review) is the author/funder, who has granted bioRxiv a license to display the preprint in perpetuity. It is made available under a CC-BY 4.0 International license.

number of human CD45+ cells, (**H**) human B cells, and (**I**) frequency of human myeloid cells 12-16 weeks after engraftment with control or *TRAC*$^{-/-}$ CD34+ HSPCs in MISTRG6 mice. (**J**) number of human CD45+ cells and (**K**) human B cells 9 weeks after engraftment with control or *CSF1R*$^{-/-}$ CD34+ HSPCs in MISTRG6 mice.

Supp Fig. 2. (**A**) PCR amplification of the *DPP9* sgRNA targeting locus. Edited cells have a smaller amplified band due to truncation deletion caused by CRISPR editing. CD34+ cells are either engrafted in vivo or kept in culture in vitro culture for 3 days. DNA from bone marrow 9 weeks after engraftment. (**B**) Gating strategy for bone marrow hematopoietic stem and progenitor populations. (**C**) Control or *DPP9*$^{-/-}$ cells are injected intrafemorally for 16 weeks, cell numbers are assessed in the blood and bone marrow.

Supp Fig. 3 (**A**) In vitro expansion culture of control and *DPP9*$^{-/-}$ CD34^{+} HSPCs. (**B**) Frequency of clones that expanded to more than 100 cells at the end of a 7-day expansion of HSCs following experimental scheme in Figure 3D.

Supp Fig. 4. (**A**) Overlay of various gene signatures of different human bone marrow populations (49) onto scRNA-seq dataset. (**B**) Expression of *AVP* and *ITGA6* encoding CD49f marking HSC populations. (**C**) Frequency of cells within each cluster for control or *DPP9*$^{-/-}$ cells. WT, control. KO: *DPP9*$^{-/-}$. (**D**) number of differentially expressed (DE) genes defined as FDR <0.1 for each cluster. HSC, MPP includes HSC/MPP-1 and HSC/MPP-2, myeloid progenitors include MDP, neu mono prog, pre DC. CLP includes CLP-1, CLP-2.

bioRxiv preprint doi: https://doi.org/10.64898/2026.06.27.735024; this version posted June 30, 2026. The copyright holder for this preprint (which was not certified by peer review) is the author/funder, who has granted bioRxiv a license to display the preprint in perpetuity. It is made available under a CC-BY 4.0 International license.

Pro B includes pro B-1, pro B-2, pro B-3. (**E**)Ingenuity Pathway Analysis of differentially expressed genes found between *DPP9*$^{-/-}$ and control HSC/MPP clusters, DE genes defined as FDR <0.1.

Supp Fig. 5. (**A**) Schematic for sequential or simultaneous double KO. (**B**) Frequency of initial cell number after electroporation. (**C**) gene knockout efficiency of *DPP9* by Sanger sequencing.

bioRxiv preprint doi: https://doi.org/10.64898/2026.06.27.735024; this version posted June 30, 2026. The copyright holder for this preprint (which was not certified by peer review) is the author/funder, who has granted bioRxiv a license to display the preprint in perpetuity. It is made available under a CC-BY 4.0 International license.

Supp Fig 1

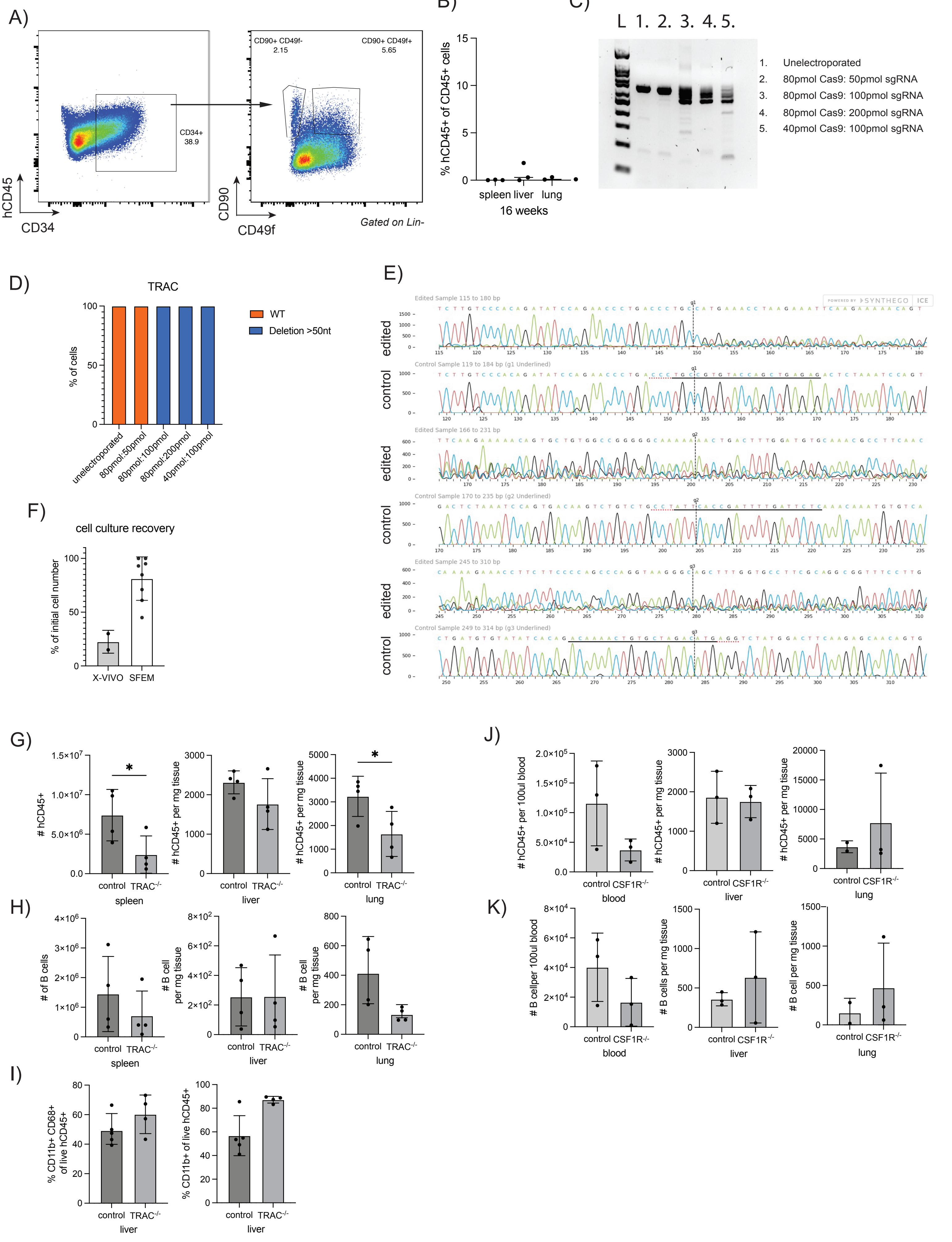

bioRxiv preprint doi: https://doi.org/10.64898/2026.06.27.735024; this version posted June 30, 2026. The copyright holder for this preprint (which was not certified by peer review) is the author/funder, who has granted bioRxiv a license to display the preprint in perpetuity. It is made available under a CC-BY 4.0 International license.

Supp Fig 2

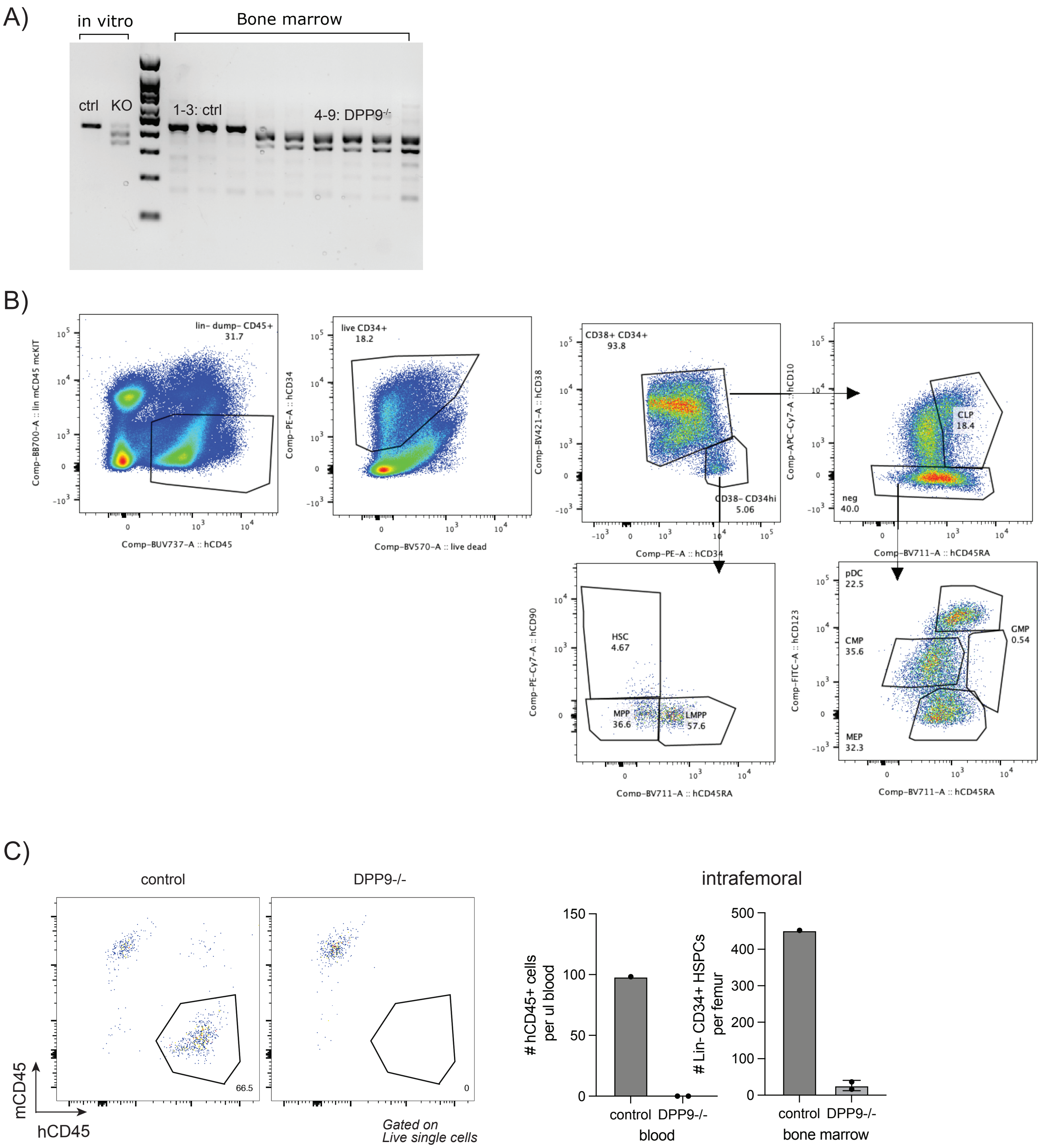

bioRxiv preprint doi: https://doi.org/10.64898/2026.06.27.735024; this version posted June 30, 2026. The copyright holder for this preprint (which was not certified by peer review) is the author/funder, who has granted bioRxiv a license to display the preprint in perpetuity. It is made available under a CC-BY 4.0 International license.

Supp Fig 3

A)

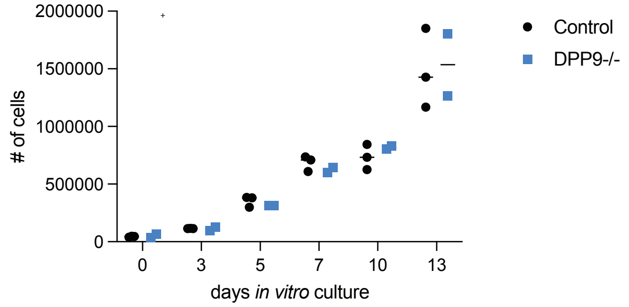

B)

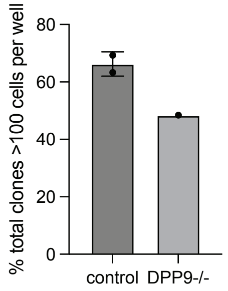

bioRxiv preprint doi: https://doi.org/10.64898/2026.06.27.735024; this version posted June 30, 2026. The copyright holder for this preprint (which was not certified by peer review) is the author/funder, who has granted bioRxiv a license to display the preprint in perpetuity. It is made available under a CC-BY 4.0 International license.

Supp Fig 4

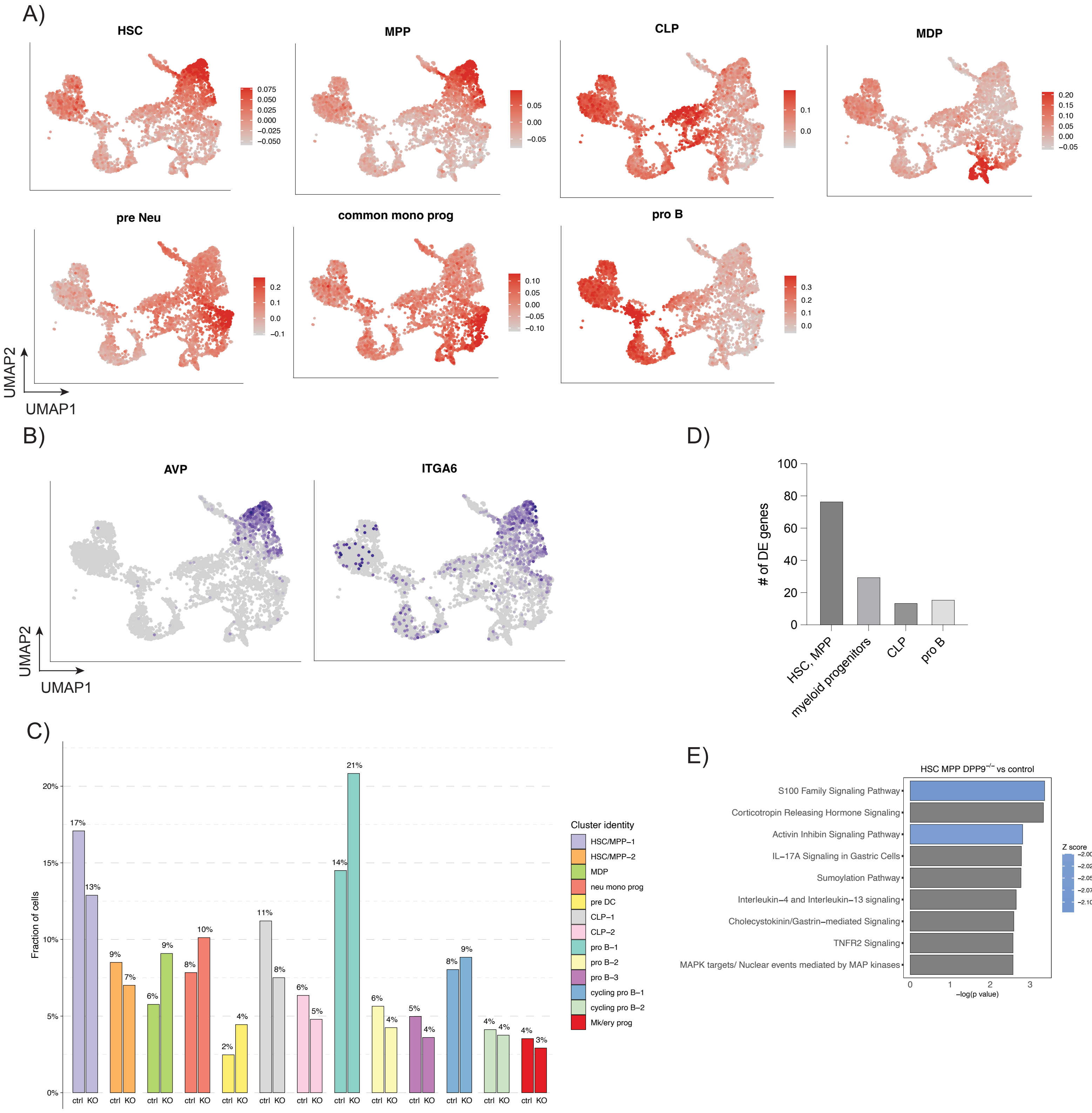

bioRxiv preprint doi: https://doi.org/10.64898/2026.06.27.735024; this version posted June 30, 2026. The copyright holder for this preprint (which was not certified by peer review) is the author/funder, who has granted bioRxiv a license to display the preprint in perpetuity. It is made available under a CC-BY 4.0 International license.

Supp Fig 5

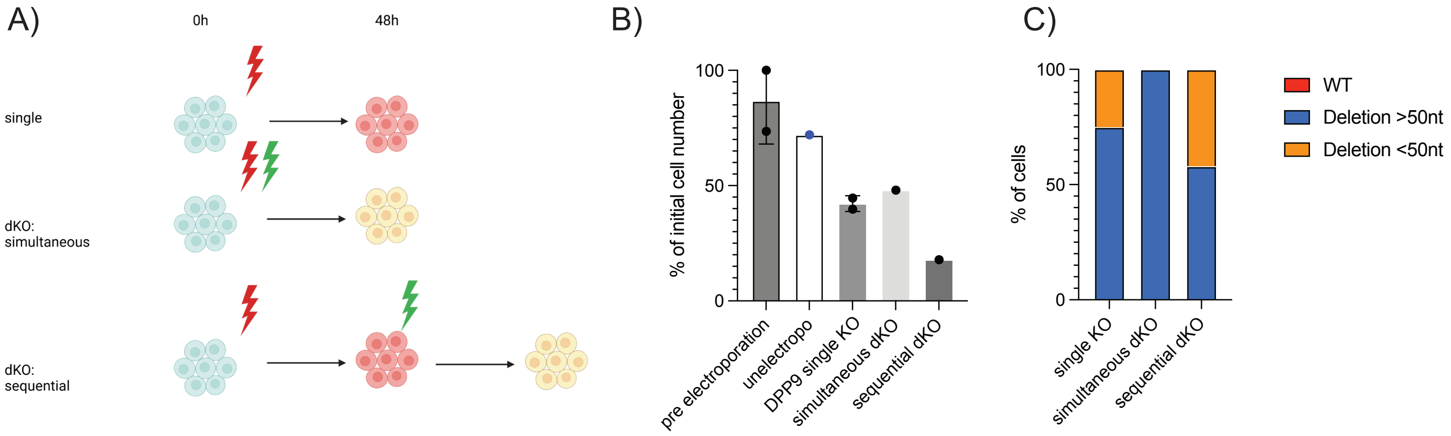

Supplement: Supplement 1 [file NIHPP2026.06.27.735024v1-supplement-1.pdf]
